# Supplementary material for: Development of a computer-aided design software for the quantitative evaluation of aesthetic damage
Source: PLoS One. 2019 Dec 18;14(12):e0226322. doi: 10.1371/journal.pone.0226322 (PMC6919621; doi:10.1371/journal.pone.0226322)
Supplement: S1 Code site — 1. (ZIP) [file pone.0226322.s001.zip › [EESC_jr][Projeto]Relatorio_07_21/reports/templates_zip/gentelella-master/vendors/select2/docs/_includes/js-source-states.html]

Alaska
Hawaii

California
Nevada
Oregon
Washington

Arizona
Colorado
Idaho
Montana
Nebraska
New Mexico
North Dakota
Utah
Wyoming

Alabama
Arkansas
Illinois
Iowa
Kansas
Kentucky
Louisiana
Minnesota
Mississippi
Missouri
Oklahoma
South Dakota
Texas
Tennessee
Wisconsin

Connecticut
Delaware
Florida
Georgia
Indiana
Maine
Maryland
Massachusetts
Michigan
New Hampshire
New Jersey
New York
North Carolina
Ohio
Pennsylvania
Rhode Island
South Carolina
Vermont
Virginia
West Virginia
